# Impairment of mitochondrial respiration in platelets and placentas: a pilot study in preeclamptic pregnancies

**DOI:** 10.1007/s11010-022-04415-2

**Published:** 2022-04-07

**Authors:** Anca M. Bînă, Oana M. Aburel, Vlad F. Avram, Theia Lelcu, Adina V. Lința, Daniela V. Chiriac, Adelina G. Mocanu, Elena Bernad, Claudia Borza, Marius L. Craina, Zoran L. Popa, Danina M. Muntean, Octavian M. Crețu

**Affiliations:** 1 Department III Functional Sciences – Pathophysiology, “Victor Babeş” University of Medicine and Pharmacy, Timişoara, Romania, Eftimie Murgu Sq. No. 2, Timişoara, Romania; 2 Center for Translational Research and Systems Medicine, “Victor Babeş” University of Medicine and Pharmacy, Timişoara, Romania, Eftimie Murgu Sq. No. 2, Timişoara, Romania; 3 Department VII Internal Medicine II – Diabetes, Nutrition and Metabolic Diseases, “Victor Babeş” University of Medicine and Pharmacy, Timişoara, Romania, Eftimie Murgu Sq. No. 2, Timişoara, Romania; 4 Department XII Obstetrics and Gynecology – Obstetrics and Gynecology I, “Victor Babeş” University of Medicine and Pharmacy, Timişoara, Romania, Eftimie Murgu Sq. No. 2, Timişoara, Romania; 5 Department XII Obstetrics and Gynecology – Obstetrics and Gynecology III, “Victor Babeş” University of Medicine and Pharmacy, Timişoara, Romania, Eftimie Murgu Sq. No. 2, Timişoara, Romania; 6 Department IX Surgery I – Surgical Semiotics I, “Victor Babeş” University of Medicine and Pharmacy, Timişoara, Romania, Eftimie Murgu Sq. No. 2, Timişoara, Romania; 7 Center for Hepato-Biliary and Pancreatic Surgery, “Victor Babeş” University of Medicine and Pharmacy, Timişoara, Romania, Eftimie Murgu Sq. No. 2, Timişoara, Romania

**Keywords:** Preeclampsia, Mitochondria, Platelet, Placenta, High-resolution respirometry, Fetal growth restriction

## Abstract

Preeclampsia (PE) is a major complication of pregnancy with partially elucidated pathophysiology. Placental mitochondrial dysfunction has been increasingly studied as major pathomechanism in both early- and late-onset PE. Impairment of mitochondrial respiration in platelets has recently emerged as a peripheral biomarker that may mirror organ mitochondrial dysfunction in several acute and chronic pathologies. The present study was purported to assess mitochondrial respiratory dys/function in both platelets and placental mitochondria in PE pregnancies. To this aim, a high-resolution respirometry SUIT (Substrate-Uncoupler-Inhibitor-Titration) protocol was adapted to assess complex I (glutamate + malate)- and complex II (succinate)-supported respiration. A decrease in all respiratory parameters (basal, coupled, and maximal uncoupled respiration) in peripheral platelets was found in preeclamptic as compared to healthy pregnancies. At variance, placental mitochondria showed a dichotomous behavior in preeclampsia in relation to the fetal birth weight. PE pregnancies with fetal growth restriction were associated with decreased in coupled respiration (oxidative phosphorylation/OXPHOS capacity) and maximal uncoupled respiration (electron transfer/ET capacity). At variance, these respiratory parameters were increased for both complex I- and II-supported respiration in PE pregnancies with normal weight fetuses. Large randomized controlled clinical studies are needed in order to advance our understanding of mitochondrial adaptive vs. pathological changes in preeclampsia.

## Introduction

Preeclampsia (PE) is the most severe hypertensive disorder of pregnancy that occurs after 20 weeks of gestation and is associated with several adverse maternal and fetal outcomes [1]. Classically defined as the increase in the systolic (≥ 140 mmHg) and/or diastolic (≥ 90 mmHg) blood pressure associated with proteinuria in previously normotensive women, PE is a systemic disorder that may involve several other organs/tissues. Accordingly, the severe forms may also present at least one of the following new-onset hematological (low platelet count, hemolysis, disseminated intravascular coagulation), renal (acute kidney injury), liver (hepatocellular injury), neurological (visual and mental disturbances, stroke) complications [2, 3].

Two forms of PE are described: *early-onset* (*EO*)* PE*, developed before the 34th week of gestation and *late-onset* (*LO*)* PE* developed after the 34th week of gestation [4]. The two subtypes show differences in terms of time of the onset, clinical presentation, biochemical markers, genetics. In the EO PE, also known as a “fetal” or “placental disorder”, the following abnormalities have been described: placental dysfunction, fetal growth restriction (FGR), premature delivery, uterine and/or umbilical Doppler, small for gestational age fetuses, other organ dysfunctions (proteinuria, low platelet count, hemolysis, disseminated intravascular coagulation, impaired liver function, acute kidney injury, pulmonary edema, neurological complications) [5]. LO PE is described more common as a “maternal syndrome”, in this form the newborns being generally normal or large for gestational age, frequent the only sign of endothelial dysfunction is the presence of proteinuria. The EO preeclampsia has an increased propensity to evolve into a severe form with adverse maternal and fetal outcomes [4, 6].

No treatment for PE currently exists, the pre-term delivery being the only known cure [7]. Albeit of elusive pathophysiology, the central abnormality in PE is the hypoxia-driven placental insufficiency. It is important to emphasize that many of the placental changes have initial adaptive roles that with the aggravation of hypoxia may become detrimental for both mother and fetus. While the abnormal placentation in early pregnancy has been associated with inappropriate trophoblast invasion by the remodeled spiral arteries that leads to endothelial dysfunction, it is the late gestational hypoxia that is mainly responsible for mitochondrial dysfunction [8] that coexists with placental hypoperfusion [9].

Mitochondria are multifunctional, dynamic organelles, central to the regulation of cellular metabolism, redox homeostasis, and cellular survival. Placenta is a metabolically active organ purported to progressively adapt and support both its own growth and the one of the fetus; mitochondria are critical in meeting the bioenergetic and biosynthetic requirements. As regarding the former, mitochondria generate ATP via oxidative phosphorylation (OXPHOS) which occurs at the level of the electron transport system (ETS) consisting of four proteins (Complexes I-IV) bound to the inner mitochondrial membrane and coupled to Complex V (ATP synthase).

Mitochondrial dysfunction has been systematically reported to be involved in the PE pathogenesis in both animal models [10, 11] and humans [12–14]. However, there is some inconsistency in the literature regarding both the impairment of mitochondrial dynamics and bioenergetics in the setting of PE. Thus, both an increase and decrease in placental mitochondrial content, fission proteins, and ETS complexes expression/activity have been reported (reviewed in refs. [4, 15]).

Platelet count decreases gradually throughout gestation both due to hemodilution and sequestration/consumption in the placental circulation. Exaggerated activation of platelets at the maternal–fetal interface has been reported to trigger local inflammasome activation and contribute to the aggravation of PE [16].

However, in the past decade there is an increasing body of research suggesting that bioenergetics profiling of platelets may inform on the presence mitochondrial dysfunction in different tissues in lieu of biopsies, in both acute and chronic pathologies [17–19]. Whether this also occurs in the setting of PE has not been investigated so far.

The present study was aimed to assess the changes in mitochondrial respiratory function in both peripheral platelets and placental mitochondria isolated from preeclamptic pregnancies as compared to healthy, non-PE pregnancies, and age-matched non-pregnant women.

## Materials and Methods

### Chemicals

All chemicals were purchased from Sigma-Aldrich.

### Platelet study

### Platelet isolation

Twenty ml of peripheral blood was collected in BD vacutainers using K_2_ (EDTA) as anticoagulant. Blood samples were prepared and analyzed within 1–4 h after collection.

A two-step centrifugation protocol at 22 °C was adapted from ref. [20]. The first centrifugation (10 min at 500×*g*) was performed in order to yield the platelet-rich plasma (PRP). The PRP was further centrifuged (5–6 min at 4600×*g*) to obtain the platelet pellet that was dissolved in approx. 1 ml homologous plasma and used for HRR (high-resolution respirometry).

### Assessment of platelet mitochondrial respiration

Respiration of isolated platelets was assessed at 37 °C, by means of high-resolution respirometry (HRR) using the Oxygraph-2 k (Oroboros Instr., Innsbruck, AT). Platelets were suspended in a mitochondrial respiration medium (MiR05): sucrose 110 mM, HEPES 20 mM, taurine 20 mM, K-lactobionate 60 mM, MgCl_2_ 23 mM, KH_2_PO_4_ 10 mM, EGTA 0.5 mM, BSA 1 g/l, pH 7.1 [21]. Calibration at air saturation was conducted before each experiment. A Substrate-Uncoupler-Inhibitor-Titration (SUIT) protocol was adapted to measure complex I- and complex II-dependent respiration.

The main respiratory parameters were as follows: ROUTINE respiration, maximal active respiration (or OXPHOS capacity, ATP-generating oxidative phosphorylation), the non-phosphorylating respiration (or LEAK respiration, in the presence of oligomycin), and the maximal uncoupled respiration (or ET capacity, in the presence of a protonophore).

In HRR experiments, cellular respiration is first allowed to stabilize at steady-state in the presence of endogenous substrates (15 min) in order to measure the ROUTINE respiration (i.e., the physiological coupling state controlled by cellular energy demands).

The SUIT protocol started after plasma membrane permeabilization (but with the maintenance of the membrane of intracellular organelles, mitochondria, and endoplasmic reticulum) with digitonin (1 µg/L × 10^6^ platelets).

Complex I-dependent respiration (CI-supported OXPHOS) was measured in the presence of malate (5 mM), glutamate (5 mM), and ADP (1 mM). Succinate (10 mM) was further injected to induce maximal OXPHOS capacity through both complex I and complex II (CI- and CII-supported OXPHOS) by allowing the convergent electron flow through CI II into the Q-junction. In the presence of conventional substrates for CI (glutamate, malate) and CII (succinate) the simultaneous, convergent electron flow into the Q-junction is mimicking the action of the Krebs cycle in the intact cell (which generates both NADH and succinate in the mitochondrial matrix). Rotenone (0.5 µM) and antimycin-A (2.5 µM) were injected to inhibit electron transfer to O_2_ (specific to the oxidative phosphorylation process).

The maximal uncoupled respiration or the ET capacity was obtained by stepwise titrations (6 μM/step) with the protonophore, carbonyl cyanide m-chlorophenylhydrazone (CCCP) until maximal respiratory flux was detected. Complex I activity was inhibited by rotenone (2 μM) thus allowing to assess the CII-supported ET capacity. The resulting slow rates of O_2_ consumption known as the residual oxygen consumption (ROX), due to processes other than oxidative phosphorylation, were subtracted from the other respiratory rates.

DatLab software (Oroboros Instr.) was used to record the oxygen concentration and oxygen consumption rate (flux) displayed in real-time and also for data analysis. Mitochondrial respiration was also corrected for oxygen flux due to instrumental background.

### Placenta study

The participants (*n* = *2*4) were randomized in 3 groups: non-PE pregnancies (*n* = 14) and PE pregnancies (*n* = 10), with (*n* = 4) or without (*n* = 6) fetal growth restriction (FGR). The intrauterine growth restriction was established through ultrasound examination with a fetal weight below the 10th percentile accompanied by the umbilical and middle cerebral artery pulsatility index [22].

Healthy placentas were harvested from non-labored term (38 ± 0.31 weeks) pregnancies with indication for cesarean delivery (previous cesarean section, fetal malpresentation, high myopia).

### Placental mitochondria isolation

In order to isolate cytotrophoblast mitochondria a differential centrifugation protocol was adapted from ref. [23]. Villous tissue (2–3 g) was collected immediately after non-labored, cesarean delivery from the maternal surface after discarding the maternal decidua; calcified areas and microinfarctions were avoided [24]. Placenta fragments were harvested from the same areas (central, medial, and peripheral regions) in each patient in order to reduce the differences caused by different sampling sites [25].

Samples were processed within 1 h after collection. Placental tissue was manually fragmented and homogenized in isolation medium: mannitol 210 mM, sucrose 70 mM, HEPES 10 mM, supplemented with 5 mg/mL BSA and 100 mM EGTA. Three successive centrifugations were performed at 4 °C (5 min/750×*g*, 10 min/10,000×*g*, and 10 min/10,000×*g*). The last sediment obtained was resuspended in 250 µL isolation medium. Prior to HRR experiments the concentration of mitochondrial proteins was assessed according to the Biuret method [26].

### Assessment of placental mitochondria respiration

Placental mitochondrial respiration was assessed by HRR using the Oxygraph-2 k (Oroboros Instr., Innsbruck, AT). The method provides the most accurate quantification of oxygen consumption in small samples via the well-established SUIT protocols that allow the sequential titration of mitochondrial substrates and inhibitors/modulators for the measurement of respiratory parameters [18].

Isolated mitochondria (0.6 mg protein/mL) were incubated in MiR06 medium (MiR05 + catalase) in the 2 mL chambers of the oxygraph. The SUIT protocol was adapted to separately assess complex I- and II-dependent respiration in chamber A and B, respectively.

For complex I, State 2 respiration was initiated by injecting the exogenous substrates, glutamate (5 mM) and malate (5 mM) into chamber A. Complex I-dependent active respiration (State 3) or the OXPHOS capacity was further initiated via the addition of ADP (1 mM), which allows protons to flow back through the ATP synthase, thus driving the process of oxidative phosphorylation (OXPHOS) and ATP synthesis. Then oligomycin (1 μg/mL) was added, to inhibit proton flow through ATP synthase, yielding the slower rate of LEAK respiration (State 4).

For complex II, State 2 respiration was initiated by injecting the exogenous substrate, succinate (10 mM) plus rotenone (2 μM) in order to inhibit CI, into chamber B. Complex II-dependent active respiration (State 3) was subsequently triggered by adding ADP (1 mM).

A cytochrome *c* test was performed to test the intactness of mitochondrial outer membrane, since in case of damage it would be released with a consequent inhibition of respiration [27]. Experiments were excluded if the increase in oxygen consumption was 10% after the addition of cytochrome c (10 μM).

For both complexes, the maximal uncoupled respiration or the ET capacity was determined by titrating the uncoupler CCCP (6 mM/titration step). The final addition of antimycin-A (1 µM)) inhibited complex III, thus allowing the measurement of ROX.

All values for the respiratory states ROUTINE, LEAK, and ET capacity were corrected for ROX.

**Fig. 1:**
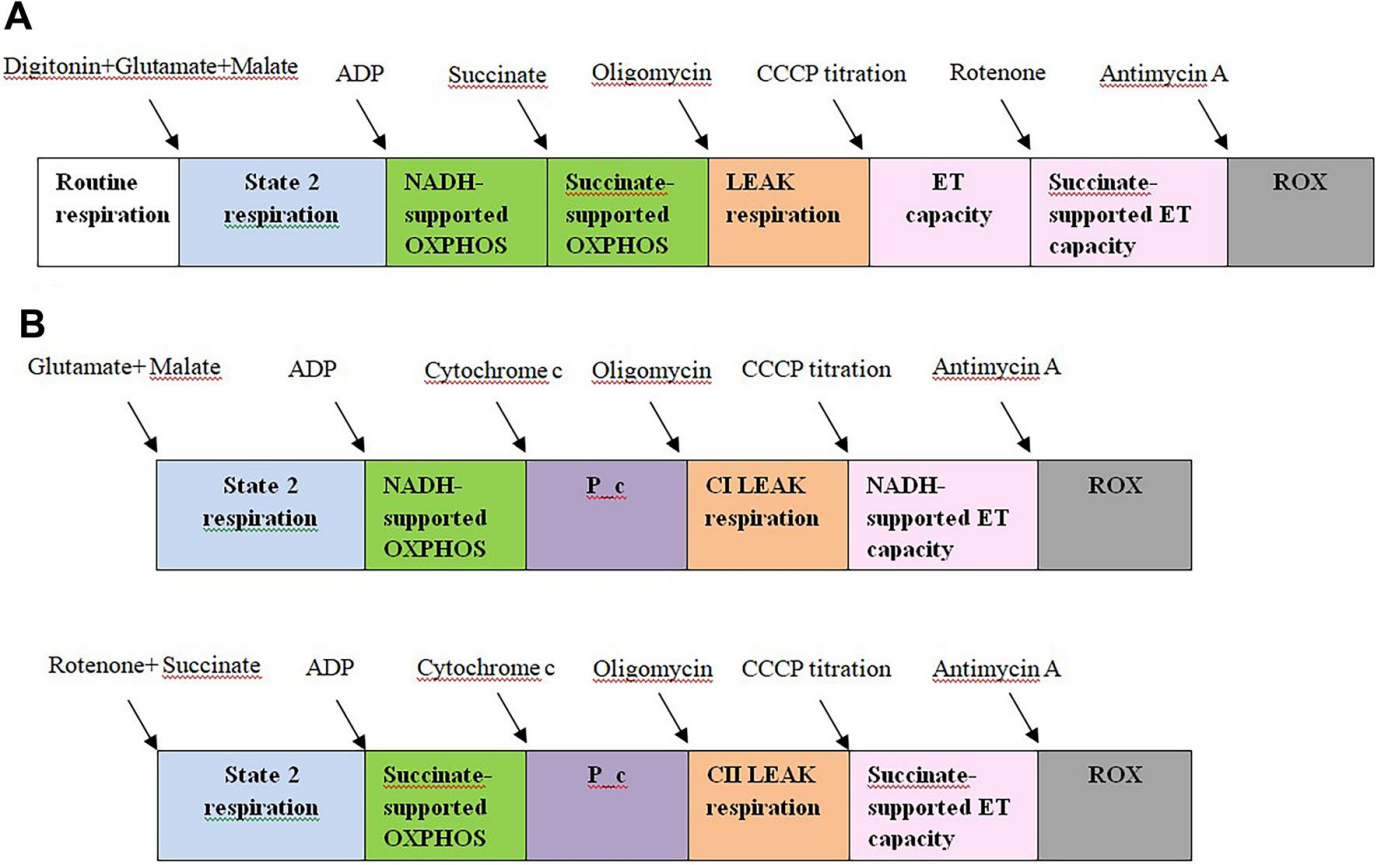
**A** Experimental protocol for the study of mitochondrial respiration in permeabilized platelets. **B** Experimental protocol for the study of respiration in isolated placental mitochondria. *ADP* adenosine diphosphate, *CCCP* carbonyl cyanide m-chlorophenyl hydrazine, *ET* electron transport, *OXPHOS* oxidative phosphorylation, *NADH* Nicotinamide adenine dinucleotide reduced form, *P_c* = OXPHOS after cytochrome *c* addition, *ROX* residual oxygen consumption

**Fig. 2:**
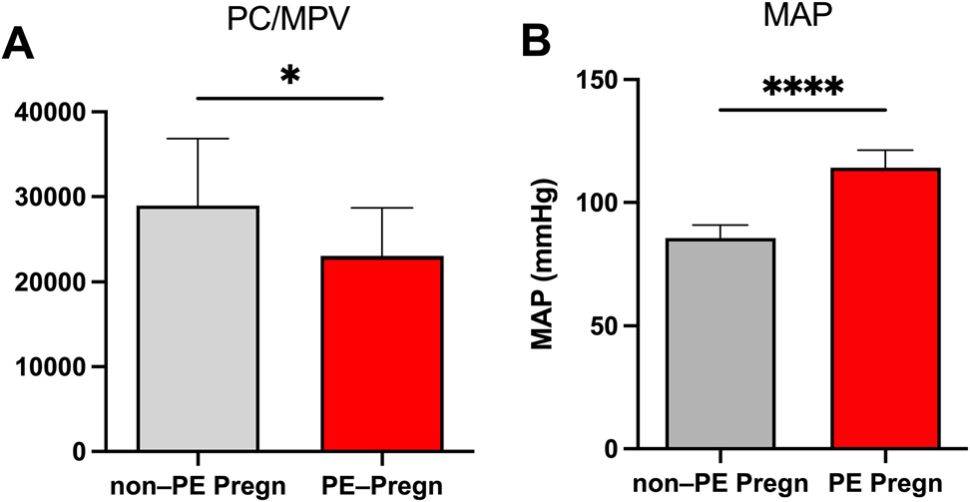
Representative trace of the SUIT protocol in permeabilized platelets. *A* non-PE pregnancy, *B* PE-Pregnancy

The experimental protocols for the HRR studies are depicted in Fig. 1A (for platelets) and B (for placental mitochondria). A representative trace of the SUIT protocol in platelets in non-preeclamptic pregnancy (A) and in preeclamptic pregnancy (B) is depicted in Fig. 2.

### Data analysis

All data are expressed as mean ± standard error of mean (SEM). To compare three groups the one-way ANOVA with Tukey’s post hoc test was performed. The student *t* test was used to determine differences between 2 groups. Significant differences are denoted *p < 0.05; ** *p* < 0.01; *** *p* < 0.001; ****p < 0.0001. Analyses were performed using GraphPad PRISM 9.01 (GraphPad, USA).

## Results

This cross-sectional, single-site study was conducted according to the Helsinki declaration (1975), as revised in 2008 and its later amendments, and was approved by the Committee for Research Ethics of “*Victor Babe*ş”  University of Medicine and Pharmacy, Timişoara, RO (no. 7 /30.01.2019 and 28/25.06.2020). All participants provided a written informed consent.

The diagnosis of preeclampsia was based on the criteria proposed by The International Society for the Study of Hypertension in Pregnancy (ISSHP), as previously defined [5]. The exclusion criteria were: diagnosis of chronic hypertension, coexistent diabetes mellitus, chronic kidney disease, twin pregnancy, inflammatory disorders, malignancies, cardiovascular disease, and coagulation disorders. All pregnant women were evaluated in the third trimester (28–40 weeks of pregnancy).

All preeclamptic patients underwent antihypertensive treatment with Methyldopa and/or Nifedipine. Patients who received antiplatelet drugs, anticoagulants or magnesium sulfate therapy were excluded from the study. Controls were healthy, age-matched non-pregnant women.

### Platelet mitochondria study

#### Study population

Participants (*n* = 33) in this study were randomized into 3 groups: PE pregnancies (*n* = 14), non-PE pregnancies (*n* = 10), and controls (*n* = 9).

#### Characteristics of the study groups

**Fig. 3:**
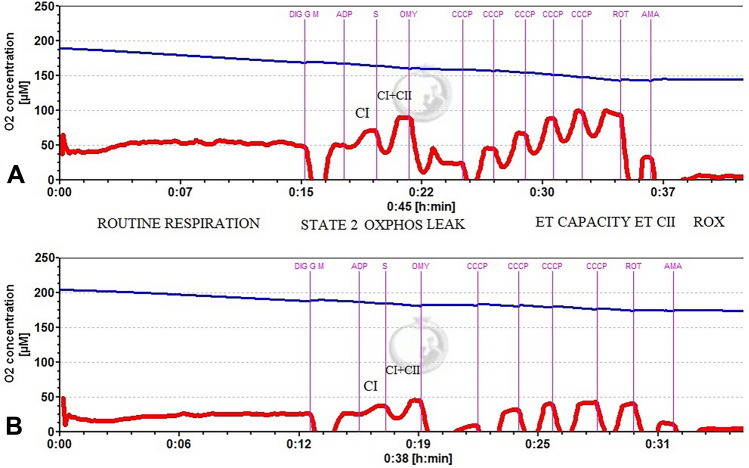
**A** The ratio PC/MPV in PE vs. non-PE pregnancies (*p < 0.05). **B** Mean arterial pressure in PE vs. non-PE pregnancies (****p < 0.0001). Data are presented as mean ± SEM. *PC* platelet count, *MPV* mean platelet volume

The following demographic and laboratory characteristics of pregnant women (PE and non-PE) were included: maternal age, gestational age, maternal blood pressure, proteinuria, creatinine, PC, MPV, leucocyte count (Table 1). Additionally, the PC/MPV ratio (Fig. 3A) and the mean arterial pressure—MAP = (Syst BP + 2 X Diast BP)/3 (Fig. 3B) were calculated in non-PE and PE pregnancies.

**Table 1:** Patients’ demographic and laboratory characteristics in the platelet mitochondria study

Parameter	PE PREGN (*n* = 14)	Non-PE PREGN (*n* = 10)	*p* value
Maternal age (years)	30.85 ± 1.04	30.71 ± 1.71	ns
Gestational age (weeks)	35.31 ± 0.84	33.71 ± 0.92	ns
Systolic BP (mmHg)	155.8 ± 2.88	112.9 ± 3.91	< 0.0001
Diastolic BP (mmHg)	94.23 ± 1.25	72.14 ± 3.76	< 0.001
MAP (mmHg)	114.2 ± 1.9	85.5 ± 1.68	< 0.0001
Proteinuria (g/24 h)	1.65 ± 0.45	–	ns
Creatinine (mg/dL)	0.72 ± 0.04	0.57 ± 0.03	< 0.05
Platelet count (μL)	209,200 ± 15,5	233,700 ± 22,9	ns
MPV (fL)	9.02 ± 0.32	8.36 ± 0.19	ns
PC/MPV	23,02 ± 2496	28,95 ± 1517	< 0.05
Leucocyte count (/μL)	11,240 ± 1,142	11,470 ± 1,096	ns
N/L	4,971 ± 0.67	4,761 ± 0.58	ns

Data are presented as mean ± SEM

*MAP* mean arterial pressure, *MPV* mean platelet volume, *PC* platelet count, *N/L* neutrophil to lymphocyte ratio, *BP* blood pressure

The peripheral platelet count (PC) and the mean platelet volume (MPV) were increased, in the in PE vs. non-PE group, albeit not reaching the statistical significance (Table 1). However, the PC/MPV ratio was significantly decreased (*p* < 0.05) in PE vs. non-PE pregnancies (Fig. 3A). A decrease in PC and/or an increase in MPV with a subsequent a decreased PC/MPV ratio have been previously has been suggested to be a predictor for the severity of PE [28, 29].

The mean arterial pressure (MAP) was significantly increased in the PE pregnancies, as the main diagnostic criterion (Fig. 3B).

#### Platelet respiration is decreased in preeclamptic pregnancies

We report here that mitochondrial respiration is significantly decreased in peripheral platelets harvested from PE vs. non-PE pregnancies.

**Fig. 4:**
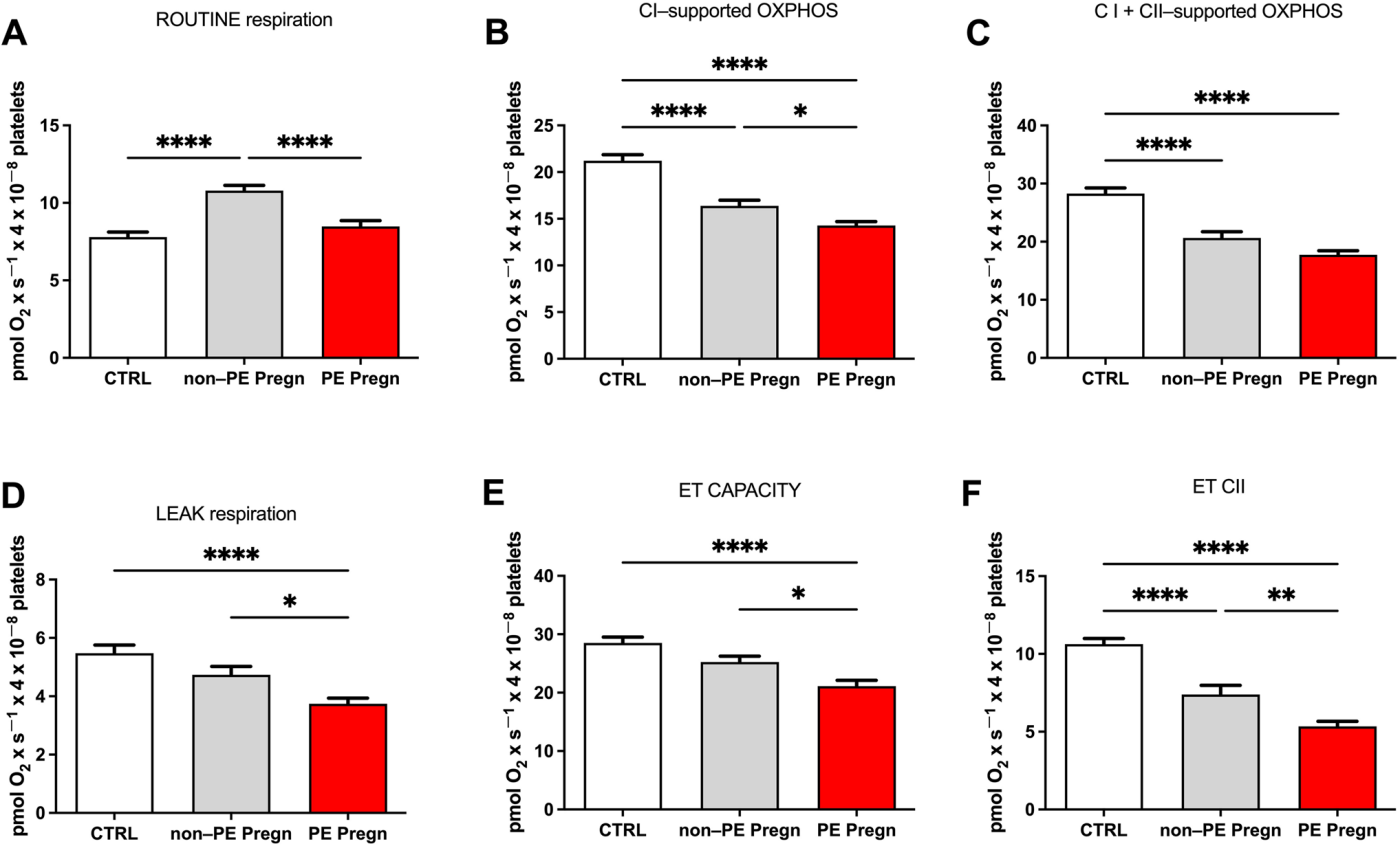
Mitochondrial respiratory parameters in permeabilized platelets. **A** ROUTINE respiration, **B** CI-supported OXPHOS, **C** CI + CII-supported OXPHOS, **D** LEAK respiration, **E** ET capacity for CI + CII, **F** ET capacity for CII. *OXPHOS* oxidative phosphorylation, *LEAK* non-phosphorylating respiration, *ET* capacity = electron transport system capacity. Data are presented as mean ± SEM

We firstly noticed a significant decrease in ROUTINE respiration in the PE group as compared to the non-PE (*p* < 0.0001) (Fig. 4A). Of note, in the non-PE group (healthy pregnancies) ROUTINE respiration, defined as the oxygen consumption of the platelet pellet in the presence of endogenous substrates, was significantly increased as compared to the age-matched controls.

Complex I-supported OXPHOS was further assessed by adding ADP in the presence of complex I substrates (glutamate and malate) and was found to be significantly decreased (*p* < 0.05) in PE vs. non-PE group (Fig. 4B). Both type of pregnancies (healthy or complicated with PE) showed more important decrease (*p* < 0.0001) in active respiration when compared to the non-pregnant age-matched volunteers (Fig. 4B), observation that was further confirmed for the complex I and II-supported OXPHOS (Fig. 4C). However, the maximum CI- and CII-supported OXPHOS, corresponding to the sum of NADH-linked OXPHOS and succinate-linked OXPHOS, showed only a minor decrease, suggesting a primary impairment of CI in peripheral platelets in the setting of preeclampsia.

The non-phosphorylating LEAK respiration, elicited by the addition of oligomycin (the ATP synthase inhibitor) was 21% lower in PE vs. non-PE pregnancies (*p* < 0.05); the decrease was even more important (36.59%, *p* < 0.0001) when compared to controls (Fig. 4D).

To determine the maximal uncoupled respiration or the electron transport (ET) system capacity (Fig. 4E), a stepwise titration was performed with CCCP (a classical uncoupler), which induces the maximum oxygen flow, the mitochondrial membrane potential being largely collapsed [30]. As for the other respiratory parameters, ET capacity was lower in PE vs. non-PE pregnancies compared (*p* < 0.05), and also significantly decreased vs. the control group (*p* < 0.0001). Finally, by adding rotenone the mitochondrial complex I is inhibited and the ET capacity dependent on complex II is determined (CII-supported ET) (Fig. 4F). In preeclampsia the values were significantly lower compared to the controls (*p* < 0.0001) and healthy pregnancy (*p* < 0.001), additionally there was a significant decrease of CII-supported ET capacity in the healthy pregnancy (*p* < 0.0001) compared to the controls.

To evaluate the extent of ET limitation by the phosphorylating system, the P/E ratio was calculated [30]. While a ratio close to 1 (P/E = 0.99) in controls indicated no limitation, in pregnant women a comparable decrease was found in non-PE group (0.81) and PE group (0.83), respectively.

**Fig. 5:**
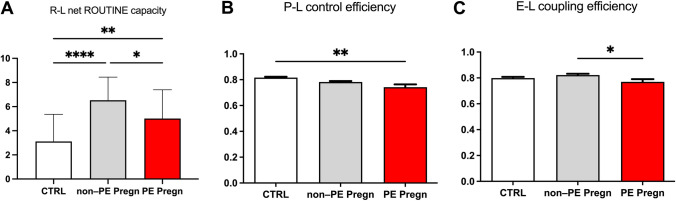
Flux-control ratios in permeabilized platelets. **A** R-L net Routine capacity calculated as the difference between ROUTINE and LEAK respiration. **B** P-L control efficiency calculated as 1-LEAK/max OXPHOS. **C** E-L coupling efficiency calculated as 1-LEAK/ET capacity. *R* routine respiration L = non-phosphorylating respiration, *P* phosphorylating capacity

R-L net routine capacity is a measure of the respiratory capacity available for the phosphorylation of ADP to ATP and was calculated as the difference between ROUTINE and LEAK respiration [30]. A significant increase of this parameter was found in the non-PE pregnancies as compared to controls (6.53 ± 0.33 vs. 3.11 ± 0.42, *p* < 0.0001). Preeclampsia decreased the parameter to an intermediate value between controls and healthy pregnancies (5.01 ± 0.4, *p* < 0.01)—Fig. 5A.

We further investigated the effects of non-PE and PE pregnancies on two flux-control ratios, P-L control efficiency and E-L coupling efficiency, calculated according to [30]. The results are presented in Fig. 5B and C. The P-L control efficiency (Fig. 5B) was calculated according to the formula 1-LEAK/OXPHOS_CI+CII_ in order to evaluate the ATP production. The normal range is between 0 and 1 and is negatively influenced by a decrease of maximal phosphorylating respiration (P) and/or by an increase in non-phosphorylating respiration (L), respectively [30]. The P-L ratio showed a comparable reduction in both types of pregnancies as compared to controls (0.81); however, a more important, albeit still minor reduction (5.12% vs. 3.7%) was found in the PE group as compared to the non-PE group. The E-L coupling efficiency (Fig. 5C) assesses the degree of coupling and was computed according to the formula 1-LEAK/ET Capacity [30]. Similarly, the coupling efficiency was significantly reduced in the PE vs. the non-PE pregnancies (*p* < 0.05).

#### Placenta mitochondria study

Participants (*n* = 24) in this study were randomized as follows: the non-PE group (*n* = 14) and the PE group (*n* = 10). The latter group was further subdivided into 2 subgroups: PE with NO FGR (*n* = 6) and PE WITH FGR (*n* = 4), in order to investigate whether differences in mitochondrial respiration occur when considering the birth weight.

In this second study, complex I (glutamate + malate) and complex II (succinate) pathways were separately examined, in line with the prior observation in the platelet study, namely the decrease in CI-dependent respiration.

We here report a dichotomous behavior of placental mitochondria in preeclampsia in relation with the fetal development: respiratory parameters were decreased in PE pregnancies associated with FGR while an increased respiration was found in PE with normal weight fetuses.

#### Characteristics of the study groups

The mean maternal age was 29.56 ± 1.78 years for non-PE pregnancies and 32.93 ± 1.08 years for PE pregnancies. The mean gestational age was 38 ± 0.31 weeks in the group of non-PE pregnancies and 35 ± 1.2 in the PE group (*p* < 0.05). Systolic, diastolic, and mean arterial pressure (in mmHg) were as follows: 123.5 ± 2.95, 73.24 ± 2.87, 83.4 ± 1.72 in the non-PE pregnancy group and 148.3 ± 2.15, 96.32 ± 1.09, and 109.3 ± 2.89 in the PE group (*p* < 0.001). The 24-h proteinuria was 2.13 ± 0.32 g in the preeclampsia group. The fetal weight at delivery was 3539 ± 106.4 g (non-PE group) vs*.* 3533 g ± 115.6 (PE with no FGR) vs*.* 2015 g ± 63.31 (PE with FGR).

The placental samples were obtained from non-labored cesarean sections, in order to prevent the previously reported influence of labor on placental mitochondrial function [31].

### Placental mitochondria respiration is globally decreased in preeclamptic pregnancies with FGR but increased in preeclamptic pregnancies with normal weight fetuses for complex I-supported respiration

**Fig. 6:**
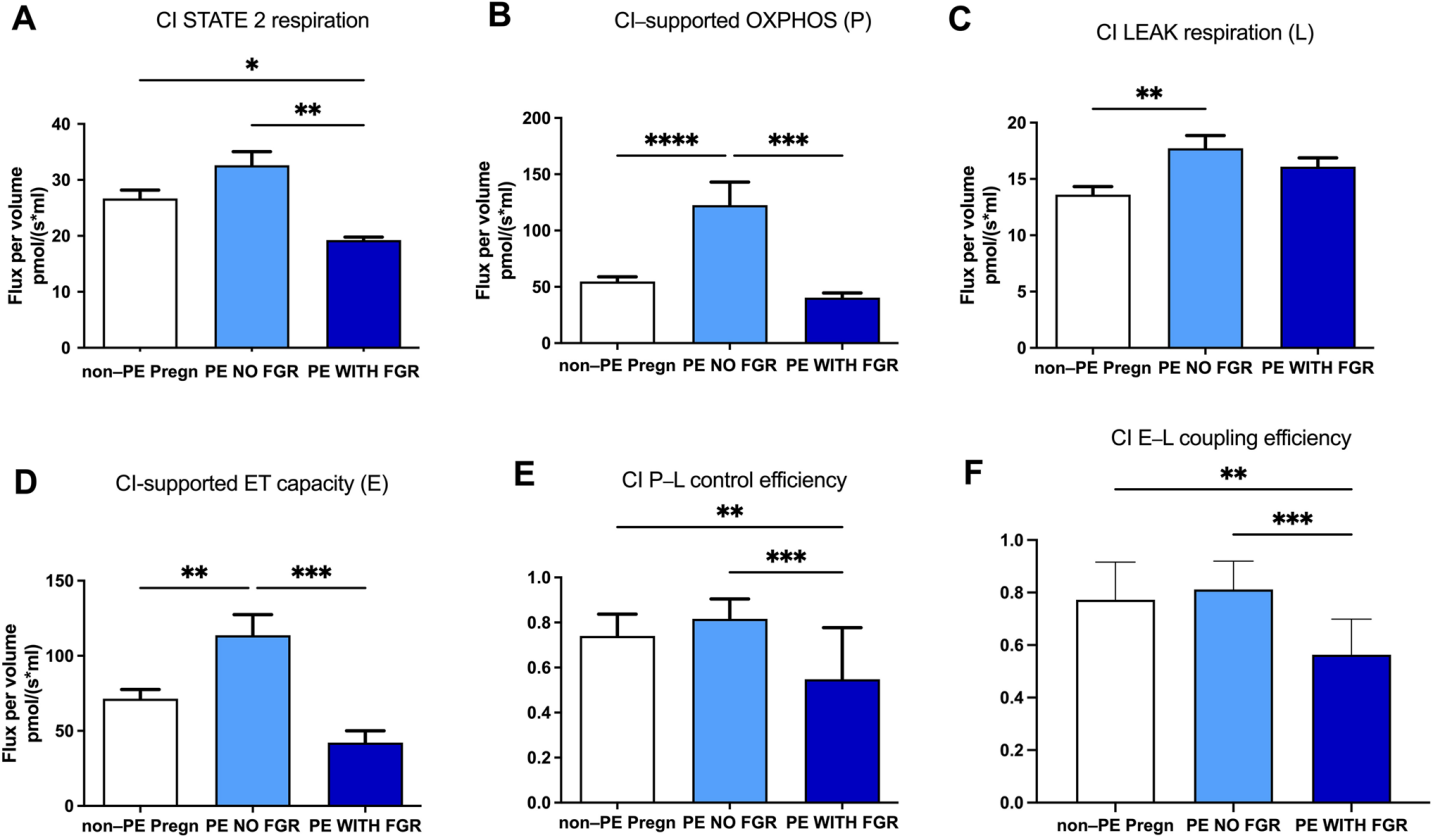
Mitochondrial respiratory parameters for complex I (CI) in isolated placental mitochondria. **A** State 2 respiration, **B** OXPHOS, **C** LEAK respiration, **D** ET capacity, **E** P-L control efficiency, **F** E-L coupling efficiency. OXPHOS (P) = oxidative phosphorylation; LEAK (E) = non-phosphorylating respiration; ET (E) capacity = electron transport capacity. P-L control efficiency was calculated as 1-LEAK/P; E-L coupling efficiency was calculated as 1-LEAK/ET capacity. Data are presented as mean ± SEM

For complex I, State 2 (basal respiration) was initiated in the presence of glutamate and malate as exogenous substrates. As shown in Fig. 6A, basal respiration was increased in the PE with no FGR, and significantly decreased (*p* < 0.05) in the PE with FGR group.

Complex I-dependent active respiration (State 3) or OXPHOS was further assessed via the addition of ADP (Fig. 6B). In the PE with no FGR group, CI-supported OXPHOS increased with 123.57% as compared to the non-PE, pregnancies (*p* < 0.0001). At variance, in the presence of FGR, the phosphorylating capacity decreased to half of the one measured in the healthy pregnancies.

Then oligomycin (the ATP synthase inhibitor) was added, yielding the slower rate of LEAK respiration (non-phosphorylating, State 4). Both types of PE pregnancies showed an increase in the non-ATP generating respiration with 30.36% in non-FGR (*p* < 0.01) and 18.3% in FGR as compared to the non-PE group (Fig. 6C).

Finally, CI-supported ET capacity or the maximal uncoupled respiration following CCCP titration showed a 59.18% increase in PE with no FGR vs. the non-PE group (*p* < 0.01). At variance, as for the maximal coupled respiration, in the presence of FGR a 41% reduction in ET capacity was noticed (Fig. 6D).

The limitation of the OXPHOS capacity by the phosphorylating system was further calculated as the P/E ratio. While the lowest value was reported for non-PE pregnancies (0.76), no limitation of the phosphorylation system affecting the CI-linked pathway was found in the PE groups, the ratio values being close to 1 (1.07 in PE with no FGR and 0.96 in FGR).

In the non-PE group, both the P-L control efficiency and E-L coupling efficiency showed comparable values (0.74 and 0.77, respectively). At variance, a very significant (*p* < 0.01) decrease in both flux-control ratios were found in the PE pregnancies with FGR vs. PE with no FGR, i.e., 0.54 vs*.* 0.81 for the former (Fig. 6E) and 0.56 vs*.* 0.81 (Fig. 6F) for the latter, respectively.

### Placental mitochondria coupled and uncoupled (but not basal) respiration is decreased in preeclamptic pregnancies with FGR and increased in preeclamptic pregnancies with normal weight fetuses for complex II-supported respiration

**Fig. 7:**
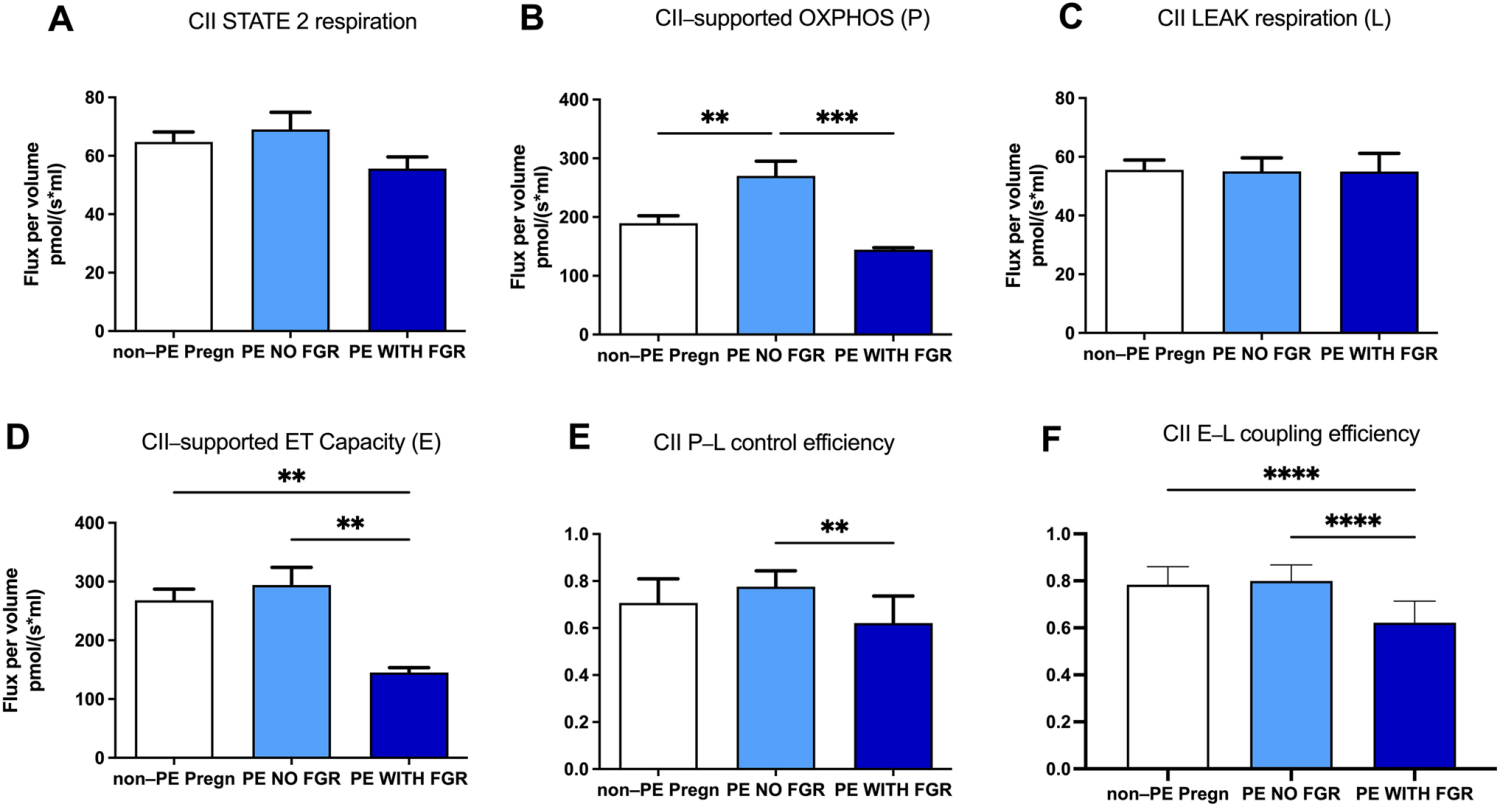
Mitochondrial respiratory parameters for complex II (II) in isolated placental mitochondria. **A** State 2 respiration, **B** OXPHOS, **C** LEAK respiration, **D** ET capacity, **E** P-L control efficiency, **F** E-L coupling efficiency. *OXPHOS *(*P*) oxidative phosphorylation, *LEAK *(*L*) non-phosphorylating respiration, *ET* capacity (E) = electron transport capacity. P-L control efficiency was calculated as 1-LEAK/P; E-L coupling efficiency was calculated as 1-LEAK/ET capacity. Data are presented as mean ± SEM

For complex II, the initiation of basal respiration (State 2) occurred in the presence of succinate as exogenous substrate (plus rotenone to inhibit the electron flow at CI). As shown in Fig. 7A, no changes in basal respiration were found in PE vs. non-PE pregnancies regardless the absence or presence of FGR, albeit a tendency of decrease was observed for the latter.

The maximal ADP-stimulated respiration (State 3) was further induced with the ATP synthesis by Complex V, the F0/F1-ATP synthase. CII-supported OXPHOS showed similar changes to the ones previously described for CI-dependent respiration, i.e., a significant increase (42.7%, *p* < 0.01) in PE with no FGR and decrease (46.5%, *p* < 0.001) in the PE with FGR group, respectively (Fig. 7B).

As showed in Fig. 7C, similarly to the results for State 2, no changes were found among the groups for the LEAK respiration (State 4) elicited after the oligomycin-mediated inhibition of the ATP synthase in order to achieve the non-phosphorylating respiration (mainly driven by proton leak from the mitochondrial intermembrane space).

CII-supported ET capacity or the maximal uncoupled respiration (following stepwise titration with CCCP) showed no change in the PE with no FGR group but a significant reduction (45.84%, *p* < 0.01) in PE associated with FGR as compared to the non-PE pregnancies (Fig. 7D).

As for the P/E ratio, similar to the CI data, no limitation of the phosphorylation system affecting the CII-linked respiration was detected in the PE groups (0.91 in PE with no FGR and 0.99 in PE with FGR), while for the non-PE pregnancies a value of 0.70 was found.

Also, as in case of CI-supported respiration, the flux-control ratios assessed for complex II were significantly reduced in the PE pregnancies associated with FGR, but not in pregnancies with normal birth weights. Accordingly, the P-L control efficiency ratio (Fig. 7E) was 0.62 in PE with FGR vs. 0.77 in PE with no FGR and vs. 0.70 in the non-PE group. Similarly, the E-L coupling efficiency ratio (Fig. 7F) was 0.60 in PE with FGR vs. 0.79 in PE with no FGR and vs. 0.78 in the non-PE group (*p* < 0.0001).

## Discussions

The present pilot study was purported to assess the mitochondrial respiratory dys/function in the setting of preeclampsia by measuring mitochondrial oxygen consumption in: (i) permeabilized platelets, and (ii) isolated placental mitochondria.

Mitochondria are the “powerhouses” of the cell due to the ATP generation through the OXPHOS process. Mitochondrial dysfunction is a central pathomechanism of organ dysfunction in several acute and chronic pathologies. Nevertheless, the invasiveness of obtaining primary human samples through biopsies is a strong limiting factor, particularly in vulnerable groups such as pregnant women. The use of circulating blood cells has recently emerged as a viable option in translational research of human mitochondrial dys/function since they can be easily obtained through a simple draw of venous blood plus they have the advantage of repeated measures over the time (for an excellent recent review see ref. [17]. In particular, assessment of mitochondrial respiration in peripheral platelets containing fully functional mitochondria has been utilized as a putative biomarker of systemic bioenergetic function [18–20].

To our knowledge, this is the first study which reports the decrease in platelet mitochondrial function using the HRR method in preeclamptic vs. healthy pregnancies. A non-significant decrease in platelet count was found in both groups of healthy (non-PE) and preeclamptic (PE) pregnancies, which may be related to a lower platelet count (PC) in peripheral blood [32, 33] or an increased platelet activation [34]; accordingly, PC did not influence the HRR experiments. An age-related difference in platelet respiratory rates has been reported in the literature, with a slightly increased ROUTINE respiration and decreased ET_CII_ with the age, but without influencing the overall respiratory function [20]. As the respiratory function of platelets in pregnancy has not been previously assessed, in order to avoid possible differences in respiratory rates influenced by age, the volunteers included in the control group were age-matched (Table 1).

Our data showed that ROUTINE respiration was significantly increased in healthy, non-PE pregnancies as compared to controls, and this increase was blunted in the presence of preeclampsia.

At variance, a significant decrease in active respiration (in particular for CI-supported OXPHOS, but also for CI- and CII-supported OXPHOS) was found in healthy, non-PE pregnancies and this decrease was further aggravated in the presence of pre-eclampsia, particularly for the CI-dependent OXPHOS. A similar behavior was also found for non-phosphorylating (LEAK) respiration and maximal uncoupled respiration (ET capacity, in particular for CII) in PE vs. non-PE pregnancies. Our results are in line with the ones reported by Malinow et al. for platelet respiration in healthy pregnancies in the 3rd trimester where all parameters were decreased when the bioenergetic analysis was performed using the Seahorse extracellular flux analyzer. At variance, these authors reported that this decline was entirely absent in platelets harvested from 3rd trimester pregnancies afflicted with preeclampsia [35]. One possible explanation for these contradictory results could be the fact that all the women with PE received infusions with magnesium sulfate (as fetal neuroprotection and antiepileptic prophylaxis); of note, magnesium has been reported to enhance the mitochondrial function due to increased ATP generation [36]. These authors also speculated that a decrease in mitochondrial number and function are part of an adaptive mechanism aimed at limiting oxidative stress, since mitochondria are a major source of cellular reactive oxygen species.

The respiratory capacity available for the phosphorylation of ADP to ATP was calculated as the difference between ROUTINE respiration and LEAK respiration (the R-L net routine capacity) [30]. A significant increase in the R-L net routine capacity was found in healthy, non-PE pregnancies; this result is, most probably, related to an increased oxygen consumption with endogenous substrates in the normal pregnancy. The adaptive response was blunted in preeclampsia, where the R-L net routine capacity was decreased.

The observation that platelet bioenergetics reflects the organ mitochondrial dysfunction and the possibility to use these cells as easily obtainable surrogates to study the age-related mitochondrial changes in skeletal muscle function [17] as well as in several pathologies, such as: type 2 diabetes [37, 38], asthma [39] have been recently reported. There is also one negative study performed in healthy young (25–35 year) women that did not support the notion that assessment of parameters of platelet mitochondrial respiration may inform on skeletal muscle mitochondrial respiration; however, these authors found the coupling efficiency of complex I in permeabilized platelets as a unique parameter that was able to qualitatively reflect the similar change in the muscle [40].

View of the clinical heterogeneity of the preeclampsia phenotypes and pathophysiology, whether the bioenergetics of circulating platelets may recapitulate the placental mitochondrial dysfunction in the setting of this complex disease needs to be confirmed in larger cohorts.

The major finding of the placental mitochondria study is a significant increase in both CI- and CII-supported coupled respiration (OXPHOS capacity) and uncoupled maximal (ET capacity) respiration in PE with no FGR as compared to PE with FGR. Specifically, for the CI-dependent respiration the above-mentioned parameters were more than double in PE with no FGR group as compared to the PE pregnancies associated with FGR. Vishnyakova et al. reported an increase in coupled (State 3) respiration in the presence of complex I substrates in the group of early-onset PE, but not in the late-onset PE group [23]. The group of Anthony Perkins also reported the increase in active respiration (State 3) dependent on complexes I and I + II and non-phosphorylating respiration (State 4) in placentas from term preeclamptic pregnancies as compared to controls [41].

Interestingly, the increase in mitochondrial respiration, namely the OXPHOS capacity via complexes I and I + II, ET capacity, and non-phosphorylating respiration has been recently reported to occur as an adaptive change in placentas harvested after laboring vaginal delivery from Tibetan women living to high-altitude (3780 m) as compared to lower-altitude (2261 m) [42]. Accordingly, it is tempting to speculate that in the setting of preeclampsia, the increase in placental mitochondrial respiration is an adaptive mechanism that occurs in pregnancies without (but not anymore in those with) FGR.

Furthermore, our data in the group of PE with FGR are in line with the results recently reported by Guitart-Mampel et al., which investigated the mitochondrial oxygen consumption in pregnancies with intrauterine growth restriction (of various causes) and reported a significant decrease of both CI-stimulated respiration and enzymatic activity in placental tissue [43]. A decrease in complexes I and II-III activities has been previously reported in placentas harvested from PE pregnancies with intrauterine growth restriction [44]. In the same vein, an early study from the group of Andrew Murray found that mitochondrial function is altered with specific suppression of complexes I and IV in cultured human placental cells subjected to hypoxia, thus potentially contributing to impaired fetal growth [45].

At variance, in their comprehensive study Vangrieken et al. reported in placenta homogenates from PE pregnancies that only the mRNA transcript levels of a mitochondrial-encoded subunit of complex IV (but not other ET complexes) were significantly lower in PE as compared to controls; this finding might be due to the fact that only 6 out of the 12 PE pregnancies had FGR. Interestingly, these authors reported a high abundance of key glycolytic enzymes, an observation suggestive of an increased reliance on glycolysis of PE placentas [14].

More recently, Vaka et al. investigated the difference in respiratory function of placental mitochondria freshly isolated from PE patients according to the gestational age, PE < 34 weeks vs. PE > 34 weeks. They reported a significant decrease in Complex I- and II-supported State 3 respiration for in both gestational ages of PE vs. non-PE controls, suggestive for a reduction in ATP production in PE mitochondria. Interestingly, the maximal ET capacity was significantly reduced only for CII-dependent respiration in the early gestational age (PE < 34 weeks) and a trend toward reduction for both CI and CII respiration was found in the in late-gestational-age (PE > 34 weeks) pregnancies. For the latter group, the authors also reported a significant decrease of both expression and activity of CIV of the ETS [13].

In our study we evaluated the placental ATP generation through the computation of flux-control ratios, the P-L and E-L coupling efficiency and showed significantly lower values for both CI and CII in the group of PE with FGR as compared to the group of PE with no FGR and the non-PE group, respectively.

Myatt et al. suggested already several years ago that long-term hypoxia or continuous cycles of ischemia/reoxygenation are responsible for mitochondrial dysfunction, decreased ATP production, and excessive ROS production [46]. In the line, the past decade has witnessed an increased interest for PE as a ‘mitochondrial disease’, a hypothesis formulated back to the late 80 s [47]. Several animal models of maternal exposure to chronic hypoxia have confirmed that gestational hypoxia leads to significant placental mitochondrial stress and adverse fetal outcomes, from decreased birth weight to developmental programming of cardiovascular and psychiatric diseases, highlighting the importance of targeting mitochondria as therapeutical approach of hypoxia-induced pathology [48, 49].

Preeclampsia is a multisystemic syndrome with and several pathomechanims such as inflammation, oxidative plus organelles (mitochondria, endoplasmic reticulum) stress, and angiogenic dysfunction contribute to its progression. While a huge amount of research has initially tackled the role impaired hemodynamics and PE origins and defined the early-onset PE phenotype (linked to defective trophoblast invasion and impaired uterine artery Doppler) vs. late-onset PE (linked to constitutional factors such as high body mass index) [50], it is currently acknowledged that preeclampsia includes several subtypes and should not be forced into a single pathophysiological model [51].

In the present study we thought to portray the mitochondrial respiratory dysfunction using an approach based on the fetal outcome in PE, namely the birth weight. An important limitation of the present study should be acknowledged: the size of the groups being reduced we did not attempt to correlate the platelet dysfunction with that seen in placental samples in order to provide unequivocal evidence that platelet mitochondria serve as a surrogate for placental mitochondria. Accordingly, a more expansive study is needed, which unfortunately is difficult to schedule due to the COVID pandemics.

## Conclusion

Preeclampsia elicited mitochondrial respiratory dysfunction in both platelets and placental tissue. We firstly reported a decrease in all respiratory parameters (basal, active, and maximal uncoupled respiration) in platelets harvested from preeclamptic pregnancies. At variance, placental mitochondria showed a dichotomous behavior in preeclampsia in relation to the fetal birth weight. PE pregnancies with fetal growth restriction were associated with decreased coupled respiration (OXPHOS capacity) and maximal uncoupled respiration (ET capacity) whereas these respiratory parameters were increased for both complex I- and II-supported respiration in PE pregnancies with normal weight fetuses. Large randomized controlled clinical studies are needed in order to advance our understanding of mitochondrial adaptive vs. pathological changes in preeclampsia.
